# Case Report: Angiotensin converting enzyme inhibitors vs. angiotensin receptor blockers in the management of chronic hypertension: a case of lisinopril-induced rhinorrhea

**DOI:** 10.3389/falgy.2024.1480569

**Published:** 2024-11-18

**Authors:** Alice A. Amudzi, Giro Richard Samale, Xavier Vela-Parada

**Affiliations:** ^1^ Department of Internal Medicine, Berkshire Medical Center, Pittsfield, MA, United States; ^2^ CHP Neighborhood Health, Pittsfield, MA, United States; ^3^ UMass Memorial Medical Center, Worcester, MA, United States

**Keywords:** angiotensin converting enzyme inhibitors (ACE-I), angiotensin receptor blocker (ARB), hypertension, lisinopril, rhinorrhea

## Abstract

A 47-year-old woman presents to our clinic with a chief complaint of rhinorrhea; she had chronic hypertension managed with four antihypertensive drugs, including an ACE inhibitor. While dry cough is a well-known side effect associated with ACE inhibitors, this case highlights a common chief complaint yet less recognized side effect of ACE inhibitors and further emphasizes the idea that overall, angiotensin receptor blockers may be a better drug of choice in hypertension due to their favorable side effect profile.

## Introduction

Lisinopril, an ACE inhibitor, effectively manages hypertension but can cause side effects such as dry cough which is well known and upper respiratory symptoms. There is ongoing debate about whether to continue using ACE inhibitors, given the comparable effectiveness of ARBs and their favorable tolerability. This case emphasizes the importance of considering alternative treatments, such as ARBs, in patients intolerant to ACE inhibitors.

## Case presentation

A 47-year-old woman with medical problems, including chronic hypertension, bipolar disorder, and substance use disorder, on methadone who presented to our clinic with a history of five years of runny nose, which she found extremely bothersome. The patient denied other symptoms such as cough, fever, facial pain, sneezing, polyps, pruritus, headaches, conjunctivitis, or sore throat. Her physical exam, including chest and EENT (Eyes, ear, nose, and throat), was unremarkable except for an erythema area from frequent nose squeezing.

Further questioning revealed that she had a sister with chronic hypertension who had rhinorrhea and nasal congestion that began a few weeks after starting lisinopril. She was evaluated multiple times at urgent care for nasal congestion with rhinorrhea with no clear etiology and provided only supportive treatment, including nasal decongestants. After several office visits by her sister's new primary care physician, on her first visit, was able to relate the beginning of her symptoms to the time around the lisinopril prescription. Hence, lisinopril was switched to losartan, after which her symptoms completely resolved. The patient also reported a similar history with her father, who experienced flushing and upper respiratory symptoms after starting lisinopril.

## Treatment & outcome

Given her strong family history and no indication for further labs, our treatment plan was to substitute lisinopril with losartan and review her case in 2 weeks. She was amenable to the treatment and followed through with her prescription, and 2-weeks later, she reported a significant improvement in her rhinorrhea and a complete resolution of her symptoms in 4 weeks.

## Discussion

Lisinopril belongs to a class of medications called ACE inhibitors used in the management of hypertension, heart failure, and diabetic nephropathy. While it is a relatively safe and efficacious drug, its side effects include dry cough (1, 2), angioedema (3–5), acute kidney injury (6), hyperkalemia (7), and oro-esophageal pemphigus (8, 9). It works by inhibiting the conversion of angiotensin I to angiotensin II to regulate blood pressure. Lisinopril may result in the accumulation of bradykinin and substance P, which increases vascular permeability and leakage of fluid from blood vessels into the surrounding tissues.

In the upper respiratory tract, this later mechanism is thought to mediate symptoms like reported postnasal drainage, rhinitis, and rhinorrhea (10, 11). Even though the exact incidence of such reported cases is unknown, there usually lies a diagnostic challenge as potential etiologies may fall under allergic, non-allergic, or both (10, 12–14). Upper respiratory tract-related illness is among the three top diagnoses in outpatient settings in the USA, and having a broad differential is crucial in providing the standard of care patients deserve and avoiding unnecessary diagnostic testing.

ACE inhibitor-induced cough is widely known by most clinicians. However, being aware of common yet less frequently recognized ACE inhibitor-induced upper respiratory symptoms such as nasal blockage, rhinitis, or postnasal drainage is essential. It is important to note that not everyone using ACE-Is will experience rhinorrhea or upper respiratory symptoms, and some studies suggest a genetic predisposition, which could have been the case for our patient, given her strong family history (15). The severity of this side effect can vary among individuals.

Also, with the increasing prevalence of hypertension (16–20), notwithstanding the decreased morbidity and mortality with ACE-I use (21), there is an ongoing debate in the research community, as well as clinical trials on whether or not to keep using ACE-Is especially since ARBs are reasonably comparable in effectiveness and may be a favorable choice in terms of tolerability and strong evidence for end-organ protection (22–25).

In summary, upper respiratory tract symptoms, including rhinorrhea, are common presenting symptoms in the outpatient, and this case highlights the importance of having a broad differential. Based on ongoing clinical evidence, we favor the use of ARBs over ACE-Is when indicated in the management of hypertension and renal disease, more so in patients who are intolerant to ACE inhibitors.

## Patient perspective

The Patient was appreciative of the medication change. Her symptoms resolved and she stated that “I feel that someone actually listened to me. Thank you. Doctor”.

## Data Availability

The original contributions presented in the study are included in the article/Supplementary Material, further inquiries can be directed to the corresponding author.

## References

[1] Shah R Segal MS Wilkowski MJ. Case report of spontaneous remission of biopsy-proven idiopathic immune complex-mediated membranoproliferative glomerulonephritis. Case Rep Nephrol Dial. (2017) 7(2):81–90. 10.1159/000477660 28868298 PMC5566695

[2] Nazir A Sheikh FM Aslam S Javaid U. Ace inhibitors. Prof Med J. (2016) 23(9):1145–8. 10.29309/tpmj/2016.23.09.1712

[3] Whitehead WJ Reid JM. Underreported risk of lisinopril-induced angioedema in a veteran population. Ann Pharmacother. (2022) 56(4):430–5. 10.1177/10600280211032404 34282637

[4] Johnson BW Rydburg AM Do VD. Lisinopril-induced small bowel angioedema: an unusual cause of severe abdominal pain. Am J Case Rep. (2022) 23:e937895. 10.12659/ajcr.937895 36413511 PMC9701529

[5] Jani C Walker A Ahmed A Rupal A Patel D Agarwal L Lisinopril induced visceral angioedema. Res Health Sci. (2021) 6(3):16. 10.22158/rhs.v6n3p16

[6] McNaughton CD Collins SP Lindenfeld J Morrison RD Daniels J Wang TJ. 40EMF outpatient lisinopril and losartan blood levels are associated with in-hospital acute kidney injury among patients with acute heart failure. Ann Emerg Med. (2018) 72(4):S19. 10.1016/j.annemergmed.2018.08.045

[7] Johnson ES Weinstein JR Thorp ML Platt RW Petrik AF Yang X Predicting the risk of hyperkalemia in patients with chronic kidney disease starting lisinopril. Pharmacoepidemiol Drug Saf. (2010) 19(3):266–72. 10.1002/pds.1923 20112435 PMC3905673

[8] Tariq U Nasrullah A Guha A Mitre M. Oroesophageal pemphigus vulgaris secondary to lisinopril use: a new side effect. Cureus. (2021) 13(4):e14333. 10.7759/cureus.14333 33972894 PMC8105187

[9] Tariq U Guha A Raja A Mitre M. S1930 lisinopril-induced oroesophageal pemphigus vulgaris: a previously unreported side effect. Am J Gastroenterol. (2020) 115(1):S1004. 10.14309/01.ajg.0000709768.07287.a5

[10] Alromaih S Alsagaf L Aloraini N Alrasheed A Alroqi A Aloulah M Drug-induced rhinitis: narrative review. Ear Nose Throat J. (2022):1455613221141214. 10.1177/01455613221141214 36377650

[11] Pinargote P Guillen D Guarderas JC. ACE Inhibitors: upper respiratory symptoms. BMJ Case Rep. (2014) 2014(jul17 1):bcr2014205462. 10.1136/bcr-2014-205462 25035451 PMC4112303

[12] Avdeeva KS Fokkens WJ Segboer CL Reitsma S. The prevalence of non-allergic rhinitis phenotypes in the general population: a cross-sectional study. Allergy. (2022) 77(7):2163–74. 10.1111/all.15223 35038765 PMC9306544

[13] Dykewicz MS Wallace DV Amrol DJ Baroody FM Bernstein JA Craig TJ Rhinitis 2020: a practice parameter update. J Allergy Clin Immunol. (2020) 146(4):721–67. 10.1016/j.jaci.2020.07.007 32707227

[14] Zicari AM De Castro G Leonardi L Duse M. Update on rhinitis and rhinosinusitis. Pediatr Allergy Immunol. (2020) 31(Suppl 24):32–3. 10.1111/pai.13164 32017218

[15] Evans WE McLeod HL. Pharmacogenomics — drug disposition, drug targets, and side effects. N Engl J Med. (2003) 348(6):538–49. 10.1056/nejmra020526 12571262

[16] Ezeala-Adikaibe BA Mbadiwe CN Okafor UH Nwobodo UM Okwara CC Okoli CP Prevalence of hypertension in a rural community in southeastern Nigeria; an opportunity for early intervention. J Hum Hypertens. (2023) 37(8):694–700. 10.1038/s41371-023-00833-x 37120682

[17] Basta K Ledwaba-Chapman L Dodhia H Ashworth M Whitney D Dalrymple K Hypertension prevalence, coding and control in an urban primary care setting in the UK between 2014 and 2021. J Hypertens. (2024) 42(2):350–9. 10.1097/HJH.0000000000003584 37796225

[18] Yamada M Wachsmuth J Sambharia M Griffin BR Swee ML Reisinger HS The prevalence and treatment of hypertension in veterans health administration, assessing the impact of the updated clinical guidelines. J Hypertens. (2023) 41(6):995–1002. 10.1097/HJH.0000000000003424 37071434 PMC10158602

[19] Ford ND Robbins CL Hayes DK Ko JY Loustalot F. Prevalence, treatment, and control of hypertension among US women of reproductive age by race/hispanic origin. Am J Hypertens. (2022) 35(8):723–30. 10.1093/ajh/hpac053 35511899 PMC10123529

[20] Dutta A Nayak G. Concerns about the prevalence estimates of undiagnosed hypertension among women aged 15–49 years in India. J Hum Hypertens. (2023) 37(6):502–3. 10.1038/s41371-021-00626-0 34650214

[21] Pinto B Jadhav U Singhai P Sadhanandham S Shah N. ACEI-induced cough: a review of current evidence and its practical implications for optimal CV risk reduction. Indian Heart J. (2020) 72(5):345–50. 10.1016/j.ihj.2020.08.007 33189192 PMC7670268

[22] Beckett VL Donadio JV Jr Brennan LA Jr Conn DL Osmundson PJ Chao EY Use of captopril as early therapy for renal scleroderma: a prospective study. Mayo Clin Proc. (1985) 60(11):763–71. 10.1016/s0025-6196(12)60418-2 3903366

[23] Sica DA Gehr TWB Ghosh S. Clinical pharmacokinetics of losartan. Clin Pharmacokinet. (2005) 44(8):797–814. 10.2165/00003088-200544080-00003 16029066

[24] Sica DA Halstenson CE Gehr TW Keane WF. Pharmacokinetics and blood pressure response of losartan in end-stage renal disease. Clin Pharmacokinet. (2000) 38(6):519–26. 10.2165/00003088-200038060-00005 10885588

[25] Dickson TZ Zagrobelny J Lin CC Ritter MA Snavely D Ramjit D Pharmacokinetics, safety, and antihypertensive efficacy of losartan in combination with hydrochlorothiazide in hypertensive patients with renal impairment. J Clin Pharmacol. (2003) 43(6):591–603. 10.1177/0091270003253367 12817522

